# Wearable PPG Multi-Sensor for Skin Humidity, Temperature, and Contact Pressure Measurement in Weak Magnetic Field Environment: First-Step Experiments

**DOI:** 10.3390/bioengineering12121361

**Published:** 2025-12-14

**Authors:** Jiří Přibil, Anna Přibilová, Tomáš Dermek

**Affiliations:** Institute of Measurement Science, Slovak Academy of Sciences, 841 04 Bratislava, Slovakia; anna.pribilova@savba.sk (A.P.); tomas.dermek@savba.sk (T.D.)

**Keywords:** contact pressure force measurement, skin humidity and temperature, wearable photoplethysmography optical sensor

## Abstract

This study describes the developed special prototype of a wearable measuring device based on a photoplethysmography (PPG) sensor. It contains also a humidity sensor and a thermometer to measure skin moisture and temperature, and a force-sensitive (FSR) element to sense a contact pressure between the measuring probe and the skin surface. All parts of the multi-sensor are shielded, to be applicable in a weak magnetic field environment. After the basic sensor’s functionality verification inside the magnetic resonance imaging tomograph, a set of experiments was performed. Comparative measurements by an oximeter confirm good correspondence with heart rate values determined from PPG (*HR*_PPG_) and FSR (*HR*_FSR_) signals—the mean absolute error lies below 0.5 min^−1^ for both types. The sensing of PPG signals on wrists was realized for Normal, Dry, and Wet skin. In comparison with normal skin conditions, drying decreases the PPG signal range by 7% and the systolic pulse width by 8%, while moistening increases the signal ripple by 3% and decreases the correlation between *HR*_PPG_ and *HR*_FSR_ values by 5%. The detailed analysis per hand and gender types yields differences between male and female subjects, while the results for left and right hands differ less.

## 1. Introduction

The monitoring of the cardiovascular system has been successfully performed for a long time by wearable optical sensors based on a photoplethysmography (PPG) principle [1,2]. This type of non-invasive, fast, and relatively precise investigation method is used not only in clinical practice [3,4], but increasingly for continual monitoring of cardiorespiratory parameters during some fitness or sporting activities [2,5] and in a lot of home entertainment applications. The shape of a peripheral pulse wave of a sensed PPG signal reflects changes in the arterial stiffness, the arterial blood pressure (ABP) [6], the heart rate (HR) and heart rate variability (HRV) [7], the pulse transmission time (PTT) [8,9], the pulse wave velocity (PWV), etc. For the detection and quantification of pain and/or stress, the Oliva–Roztocil index (ORI) [10] has been effectively used. All these biological parameters can be further used for detection of the stress effect [11,12] from one or multi-channel PPG signal(s) [7,13].

Heart structure and function can be examined by a technique based on magnetic resonance imaging (MRI): cardiovascular MRI [14]. The scanning device using this non-invasive investigation technique generates some mechanical vibration and acoustic noise [15] with an impact on the mental and physiological state of the tested person [16]. The intensity of the vibration and level of noise generated by MRI equipment depend on several factors. These comprise the used physical principle of the generation of the free induction decay (FID) signal and the chosen class of the corresponding scan sequence (gradient or spin echo). Also, the methodology of MR image construction from the received FID signals (standard, turbo, hi-resolution, 3D, etc.) together with the basic parameters of MR scan sequences (repetition time—TR, echo time—TE, slice orientation, etc.) have significant effects on final produced vibration and acoustic noise. The settings of additional parameters such as the number of accumulations, number and thickness of slices, etc., depend mainly on the required final quality of MR images.

The electrocardiography (ECG) signals cannot be principally measured inside the running MRI device due to strong radiofrequency (RF) disturbance together with the stepwise-changed magnetic field via a gradient system. Therefore, we started our investigation by testing several commercial fitness bracelets, smart watches (working in a reflectance mode), and pulse oximeters (operating in a transmission mode). The main disadvantage of these types of devices is the fact that they are not designed for function in requested experimental conditions. They usually consist of small pieces from ferromagnetic materials, which can interfere with a magnetic field of an MRI tomograph. Their use inside the scanning area of the MRI device is very problematic without special (additional) shielding of all electronic parts. First of all, unprotected electronic circuits may be destroyed, or the device may temporarily stop working. Mainly, the display part is influenced by the presence of a magnetic field; its functionality is restored after removing it from this environment. For this reason, we finally decided to develop new PPG sensor prototypes which could solve these problems and limitations. Before its use in real measuring experiments, the sensor’s functionality as well as its wireless Bluetooth (BT) connection and the stability of bi-directional data transfer with the control device must be checked first.

In previous works, we have applied methods based on PPG wave properties for the detection and quantification of the stress level during examination in the MRI device working with a low magnetic field [17]. It is in correspondence with the final task—to find proper settings of mentioned MR scan parameters that minimize generated vibration and acoustic noise, and in this way decreasing the stress factor induced on an examined person but preserving high quality of MR scan images.

The current work was motivated by need for practical recommendations for planned long-time measuring experiments based on wearable PPG sensors in the low magnetic field environment. First, it is necessary to detect, evaluate, and quantify possible changes in PPG wave properties, affected by different levels of skin humidity, temperature, and applied contact pressure, on the temporal features of sensed PPG waves.

This paper describes the realization of a special prototype of a wearable PPG multi-sensor with an integrated I2C humidity meter and a thermometer to carry out the measurement of a skin condition at the position of an optical sensor. The developed sensor is supplemented with a force-sensitive resistor (FSR) element enabling the measurement of the contact pressure between the PPG sensor probe and the skin surface. Due to its planned use in experiments inside the scanning area of the MRI tomograph also the current sensor prototype consists of non-ferromagnetic materials, and all parts are shielded by aluminum boxes. After verifying the sensor’s functionality in laboratory conditions, the stability and quality of wireless Bluetooth (BT) connection was tested in the environment of the scanning MRI device and the received signal strength indicator (RSSI) parameter was measured. Then, the conductance/pressure conversion characteristic of the used FSR component was measured, and linearization based on the linear regression approach was applied to enable simple implementation into the multi-sensor’s control program for the real-time pickup of all four parameters. Next, comparative measurements with the blood oximeter device were performed for the calibration of the determined HR values. The main measuring experiment consisted of PPG signal sensing on a wrist with different levels of skin moisture (by washing a hand, by drying a skin, etc.) before each of the measuring phases. The database of sensed data records collected in this way was next statistically analyzed.

The main highlights of the present study can be summarized as follows: (1) the developed prototype of a wearable multi-sensor enables the real-time sensing of the PPG signal and contact pressure, and it also measures the skin moisture and temperature; (2) the constructed multi-sensor is suitable for measurements in conditions of a low magnetic field with RF distribution, which exists in the scanning area of the running MRI tomograph; (3) the realization of a PPG multi-sensor represents a relatively simple low-cost solution using only standard commercial products (no special chips or components), but functional and proper for our current experimental purpose; (4) apart from the pressure force measurement, the implemented FSR element is also usable for sensing heart pulses on a wrist radial artery—the obtained HR values and pulse wave properties are fully comparable with those determined from the PPG signal; (5) comparative measurement with a commercial oximeter shows good agreement between the determined HR values; (6) the influence of drying/moistening skin manipulations on the temporal features of the sensed PPG wave was confirmed experimentally; and (7) changes in humidity, temperature, and PPG signal properties measured by this multi-sensor can be next used for detection and quantification of the stress level of an examined person during scanning inside an MRI device.

## 2. Related Works

Principally, the optical PPG sensor can work in a transmission or reflectance mode. The transmission type of the sensor probe usually has the form of a finger ring with a light source (one or more LED elements) and a photo detector placed on opposite sides of the sensed human tissue—this realization is mainly applied in pulse oximeter devices. In the case of the reflectance type, a photo detector measures the intensity of the light reflected from the skin and it is placed on the same side of the skin surface as a light source transmitter. The reflectance PPG sensors are worn typically on the fingers or wrists as a part of wearable devices, such as fitness bracelets, smart watches, etc. [18]. In both types, the picked-up PPG signal contains two local maxima representing systolic and diastolic peaks that provide valuable information about the pumping action of the heart. Three types of sensed PPG signals can be classified: a raw pulse wave, and its first/second derivative (FD/SD-PPG). While the raw PPG signal does not seem suitable for local maxima determination, the first-order and the second-order derivatives are more informative due to more pronounced local extrema [7]. Therefore, for the purpose of the PPG pulse wave analysis, the PPG signal derivatives are generally calculated. The amplitude of the sensed PPG signal is usually not constant but is modulated, and it can often be partially disturbed or degraded. For this reason, some de-trending and filtering operations must be applied on the picked-up PPG signal before its analysis [19]. Multi-channel PPG signals picked up simultaneously by sensors mounted on different parts of the arms (hands) or legs (feet) have been used for this purpose for a long time [20]. Commercial, wearable PPG sensors use typical sampling frequencies of between 50 and 100 Hz [2]. In continuous long-term screening and monitoring, sampling at lower frequencies is also applicable (e.g., in the case of a wrist-based wearable device for atrial fibrillation detection) [4]. On the other hand, a higher sampling rate (more than 500 Hz) is justified for analysis of the systolic pulse with higher accuracy, and it is also important for the precise determination of ORI, PTT, PWV, or other PPG wave parameters. At present, special wearable smart devices used for monitoring multiple vital parameters contain sensors for the continual acquisition of ECG and PPG signals [5,21]. In all cases, it holds that the quality of the sensed PPG signals and the precision of the determined PPG wave parameters depend on the position of the optical sensor and the current state of the skin surface, including its humidity, temperature, and other factors [22].

Sensors for the measurement of the skin temperature and humidity work via different physical/electro-chemical principles [23,24,25,26]. Humidity sensors are usable for detection and evaluation in different areas of human life including respiratory behavior, skin moisture, and diaper monitoring, etc. [27]. In addition, this type of wearable sensor is helpful for application in different tasks oriented to human communication—such as in speech recognition, for emotional mode recognition, and recently it has been implemented in a non-contact human–machine interface [28,29]. Changes in skin moisture (humidity) as well as temperature can be induced by stress. Therefore, together with HRV and blood pressure parameters, it is often used for the detection and quantification of the stress intensity [30]. In this case, the sensors for measuring the electrical conductance of skin (galvanic skin response—GSR) are usually applied to map changes in skin moisture [31,32]. However, they cannot operate safely and reliably in an environment of a running MRI device, so it is practically impossible to use them in our experimental conditions.

Also, the contact pressure force exerted in the place of the sensor affects the shape of the sensed PPG wave, as confirmed by performed experimental studies [33,34]. Practically it holds that higher or lower pressures exerted by the probe lead to decreased amplitude caused by the collapsing and fluttering of vessel walls. The pressure sensor based on force-sensitive resistors (FSR) is practically usable in many biomedical applications [35,36]. For our experimental purpose, the FSR component is applicable to measure the heart pulsation on the wrist radial artery and to perform comparison with HR values determined from the PPG signal [37,38]. Finally, the ORI feature represents the ratio of the systolic pulse width and the heart pulse cycle duration, so it can be used as a representative parameter for mapping the pressure effect.

## 3. Methods

### 3.1. PPG Signal Description and Features Determination

**Figure 1:**
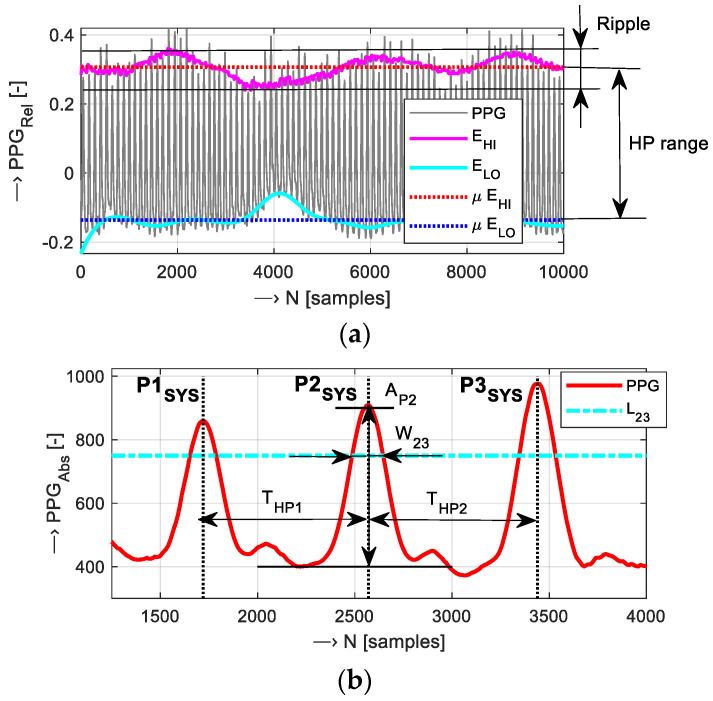
An example of PPG signal processing: (**a**) 10 k sample part of an PPG signal with calculated envelopes *E*_HI_ and *E*_LO_ and their determined mean levels, together with the denoted signal range and ripple, and (**b**) detailed part with localized three systolic peaks (vertical dashed lines) and description around the pulse *P*2_SYS_ with the denoted amplitude (*A*_P2_), the pulse width *W*_23_ determined at the level *L*_23_ (at the height of 2/3 from the offset), and adjacent pulse periods *T*_HP1,2_.

Some temporal and energetic features of the PPG wave are usable in the description of PPG signal properties and stress condition recognition [7]. Three types of HR values were used in the current task: those determined from the sensed PPG waves (*HR*_PPG_), calculated from the pressure signal sensed by an FSR element (*HR*_FSR_), and measured by an oximeter device (*HR*_OXI_) within the calibration part of experiments. The PPG wave properties are determined as follows: the upper and lower envelopes (*E*_HI_, *E*_LO_) are calculated by the low pass filtering of the squared input signal. In the case of the SD-PPG wave, the *E*_HI_ corresponds with the systolic peaks, and the *E*_LO_ with the diastolic ones—see Figure 1a. While the absolute difference between the *E*_HI_ and *E*_LO_ mean values represents the PPG signal range, *HP*_RANGE_ = *μE*_HI_ − *μE*_LO_, the heart pulse ripple in percentage is calculated as *HP*_RIPP_ = ((max *E*_HI_ − min *E*_HI_)/*μ HP*_RANGE_) × 100 using the maximum and minimum values of the upper envelope *E*_HI_ and the mean signal range. Then, the localized peak positions *P*_SYS_ are applied to determine the heart cycle periods *T*_HP_ (in samples), and using the sampling frequency *f*_S_ in Hz, the heart rate in min^−1^ is evaluated as *HR* = 60 × *f*_S_/*T*_HP_. The ORI parameter is generally defined as a ratio of the systolic pulse width and the pulse cycle duration (at the height of 2/3 from the offset—*W*_23_) [10], as depicted in Figure 1b. For the evaluation of ORI/HR sequences, the variability of ORI/*HR*_VAR_ in terms of their percentages can be calculated using the mean (*μHR*) and the standard deviation (*σHR*) values as *HR*_VAR_ = *σHR*/*μHR* × 100.

**Figure 2:**
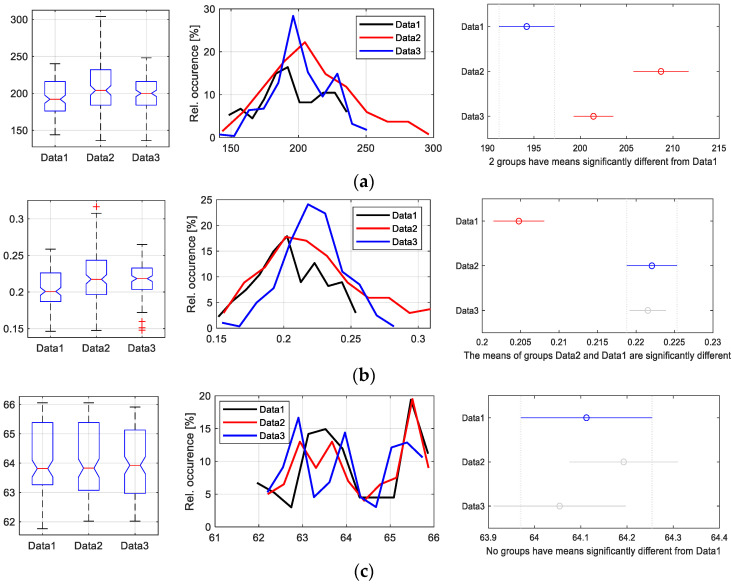
An example of extended statistical analysis—box plots of basic statistical parameters (**left**), histograms of value distribution (**middle**), visualization of multiple comparison of group means applied to the results of ANOVA statistics (**right**) for a situation when (**a**) each group of Data1, Data2, and Data3 is significantly different and also their intervals are disjoint, (**b**) when the means of groups “Data1, Data2” and “Data1, Data3” are significantly different and their intervals are disjoint, while groups “Data2” and “Data3” are not significantly different and their intervals overlap, and (**c**) when all three groups are not significantly different and their intervals overlap.

While the parameters describing the PPG signal properties are determined as one value for the whole PPG wave, the HR, as well as the W_23_ and the ORI values, are calculated for each of the detected HR cycle periods *T*_HP_. For this reason, the first group of parameters contained only the basic statistical values such as mean, minimum, maximum, and standard deviation. In the case of the second group of parameters, many more statistical values were obtained for further processing and analyses. Therefore, the extended statistics, including the histograms of value distribution and the analysis of variances (ANOVA), can be applied here [39]. This approach is focused on testing whether there is a common mean of several groups of parameters (produced by different skin manipulations). The results of the ANOVA statistics (ANOVA F-test gives the ratio of variances between and within groups [40]) can be next used for visualization based on the multiple comparison of group means technique together with box plots of basic statistical parameters and histograms, as shown in an example in Figure 2. Here, each group mean (from Data1, Data2, and Data3 sequences) is represented by a symbol and an interval around the symbol. The upper set of graphs in Figure 2a illustrates the ideal case, when all three groups, Data1, Data2, and Data3, are significantly different, and their intervals are also completely disjoint. Next set of graphs in Figure 2b represents the situation when the group means of “Data1, Data2” and “Data1, Data3” are significantly different (their intervals are disjoint), while groups “Data2” and “Data3” are not significantly different (their intervals overlap). The last set of graphs in Figure 2c shows the worst case, when all three groups are not significantly different and their intervals overlap. For creation of this example, three data sequences of several parameters obtained during current experiments were consecutively used. They were determined from PPG signals sensed on the left hand of a male tested person for three investigated skin moisture levels. In the first case, the values of the *W*_23_ parameter (in samples) were used, for the second example the values of the ORI feature were used, and the last example was created with the help of the HR values. These datasets of parameters were selected to clearly demonstrate the described evaluation by the approach based on ANOVA statistics.

Finally, the HR values obtained from the PPG and FSR signals can be compared with those measured in parallel by an oximeter device. We evaluate the accuracy of the HR determination process by the simple absolute difference *HR_DIFF_* = *HR*_OXI_ − *HR*_PPG/FSR_ in min^−1^. Its mean value (*μ HR_DIFF_*) is used to enumerate the mean absolute error (MAE) parameter for the purpose of further comparison. The attained MAE values are then analyzed—the basic statistical parameters and histograms are calculated.

### 3.2. Sensing and Analysis of Humidity and Temperature Values

Humidity sensors operate on the principle of change in the electrical impedance (capacitance) with varying moisture levels. The sensor’s active component typically contains a hygroscopic substance, i.e., a material that absorbs water or water vapor [41]. Changes in a dielectric constant generated in this way are detected and expressed as difference in relative humidity (RH in %). Some prototypes of humidity and temperature sensors were developed for special experimental purposes in biomedical research [26,42], and many sensors for skin moisture measurement work on the GSR principle [31,32]. For non-contact skin moisture investigation, the air humidity sensors are also applicable. An easy way is to use a commercial monolithic chip with integrated humidity and temperature sensors which include also an analog-to-digital converter (ADC) and an I2C interface. This chip next performs signal processing, data calibration including polynomial non-linearity correction, and hysteresis effect minimization [43]. Therefore, each time the RH measurement is made, the temperature is measured too, for the purpose of temperature compensation. It means the temperature T1 values are at disposal in parallel with the RH ones.

**Figure 3:**
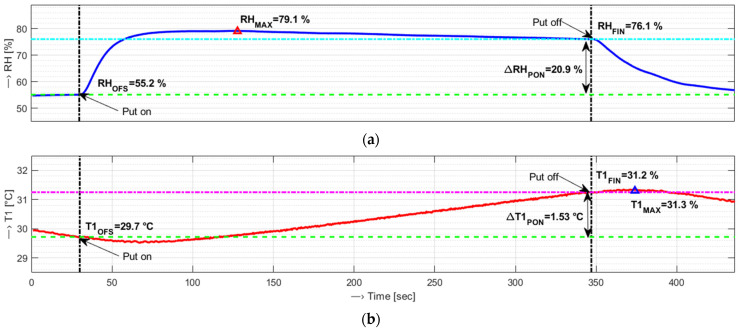
Visualization of relative humidity and temperature changes during the whole experiment with denoted time instants of putting the measuring probe on and off (vertical dash-dot lines), determined offset (green dashed lines), final (cyan/magenta lines}, and maximum values for: (**a**) RH sequence together with calculated span change, ∆*RH*_PON_, and (**b**) temperature T1 sequence including ∆*T*1_PON_ value.

To map relative humidity and temperature changes when the sensor’s probe is mounted on the skin of the testing person, the differences ∆*RH*_PON_ and ∆*T*1_PON_ can be used. These parameters are calculated as difference values between the time instants of putting the measuring probe on and off, as can be seen in an example in Figure 3. It means that the offset values *RH*/*T1*_OFS_ (which represent background humidity and temperature values) and the final *RH*/*T1*_FIN_ values (sensed just before the sensor’s probe is put off) are defined to calculate the whole span changes Δ*RH*_PON_ and Δ*T1*_PON_ as a simple subtraction: *RH*/*T1*_FIN_ − *RH*/*T1*_OFS_. Also, the maximum achieved humidity and temperature levels *RH*_MAX_ and *T1*_MAX_ can be used for further comparison and evaluation.

### 3.3. Contact Pressure Determination Using the FSR Element

**Figure 4:**
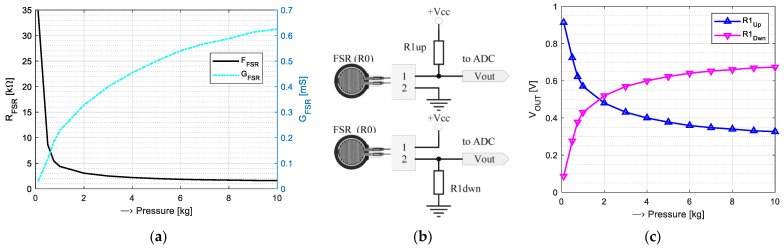
Calibration process: (**a**) example of resistance/conductance vs. pressure relationship of an FSR component [45]; (**b**) typical wiring diagrams of a voltage divider using R1 as push-up (R1up) and pull-down resistor (R1dwn); and (**c**) corresponding *V*_OUT_ curves.

From a physical point of view, the FSR element is realized in the form of a flexible thin-film pressure resistive sensor. The output resistance decreases as the pressure on the sensor surface increases [44]. There is typically a non-linear relationship between the output resistance *R*_FSR_ in kΩ and the applied pressure in kg. Therefore, in practice, the conductance *G*_FSR_ = 1/*R*_FSR_ is usually used for building of the pressure characteristic—see an example in Figure 4a.

For pressure measurement based on the FSR component, the voltage divider is usually applied. Its supplementary resistor R1 can be wired either as a push-up component connected to the power supply, or as a pull-down resistor connected to the ground—see the wiring diagram in Figure 4b. The output analog signal *V*_OUT_ from both types of voltage dividers (see examples in Figure 4c) depends on the actual value after A/D conversion (*V*_ADC_), the resolution *AD*_RES_ of the used ADC, and the applied powering voltage *V*_CC_ as *V*_OUT_ = *V*_ADC_ × *V*_CC_/*AD*_RES_. When the R1 is in the role of a push-down resistor, the resistance of the FSR element is *R*0 = *R*1 × (*V*_CC_/*V*_OUT_ − 1), otherwise *R*0 = *R*1 × (1 − *V*_CC_/*V*_OUT_).

The mostly used Arduino-compatible boards are based on processors of the ATmega328 or ATmega16/32U4 series with integrated ten-bit ADCs. In this case, *AD*_RES_ = 2^10^ = 1024, and therefore the analog signal from the FSR sensor is digitalized to 1024 levels (the numerical range is 0–1023). In practice, processors with eight-bit ADCs are also applicable, so the analog signals can be digitalized to the maximum of 256 levels, but using this lower numerical range causes decrease the accuracy and clamping of the measured pressure/resistance characteristics. The ADCs with a minimal resolution of 16-bits must be used to obtain high-precision pressure/resistance conversion characteristics. It brings some practical difficulty; an addition external ADC chip (for example the ADS111x series by Texas Instruments) should be serially connected to the main micro-controller board via an I2C bus, which increases the time necessary for A/D conversion and it limits the maximum sampling frequency in the final effect.

Before the implementation of an algorithm for the determination of the contact pressure (C*P*) in grams using *G*_FSR_ values in mS, some linearization of the depicted FSR’s conversion characteristics is necessary. To evaluate the linearization accuracy, the differential parameter *CP_LDIF_*, defined as *CP_LDIF_* = *CP*_MEAS_ − *CP*_LIN_, can be applied, where *CP*_MEAS_ represent pressure values from the measured *G*_FSR_ curve, and *CP*_LIN_ are values obtained by the linearized characteristic. These differences are next statistically analyzed to obtain minimum, maximum, mean (*μ CP_LDIF_*), and standard deviation—std (*σ CP_LDIF_*)—values.

**Figure 5:**
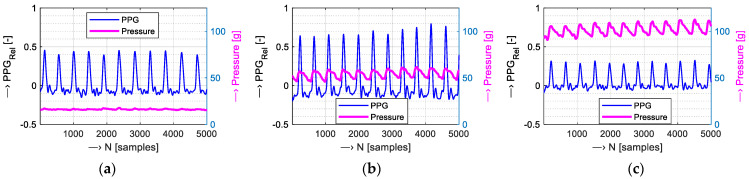
Example of a 5 k sample PPG wave with a pressure signal measured in parallel by an FSR element: (**a**) with a pressure level that is too low—mean CP = 15 g; (**b**) with the proper pressure value—mean CP = 55 g; and (**c**) with a pressure that is too high applied—mean CP = 110 g. All signals taken on the left wrist of a female testing subject, *f*_s_ = 500 Hz.

The FSR element can also be used for the sensing of the blood pulsation in a vessel (on a wrist), and it can be compared with PPG signals based on an optical principle. When a sufficient contact pressure of the PPG sensor is applied to a skin vessel, a pulsation in the rhythm of systolic peaks of the PPG wave is detectable (see an example in Figure 5b). On the other hand, a pressure that is too low generates a PPG signal with lower range, and the diastolic pulse part is not well expressed (see the graph in Figure 5a), while too strong an applied pressure can degrade and compress the PPG signal taken from a vessel, as documented by the graph in Figure 5c. In this way, HR values (*HR*_FSR_) and FSR signal parameters (range—*FSR*_RANGE_, mean—*μ FSR*, and std—*σ FSR* values) can be determined next. To map the correlation between heart rates determined from the PPG signal (*HR*_PPG_) and the *HR*_FSR_ values, the Pearson correlation coefficient *Rcc* can be calculated, and the scatter plot can be used for visualization.

## 4. Experiments

### 4.1. Structure and Realization of a Wearable PPG Multi-Sensor

The currently realized prototype of a wearable PPG multi-sensor (also called “PPGs-HTF”) consists of two basic parts: the measuring probe and the sensor’s body with a battery cell for powering. The measuring probe contains the following components:

1. The reflectance optical PPG sensor with fully integrated analog interface: the Pulse Sensor Amped (Adafruit 1093) [45] by Adafruit Industries, New York, NY, USA;
2. The Adafruit Si7021 Temperature & Humidity I2C Sensor (Adafruit 3251—STEMA QT) [43] by Adafruit Industries, New York, NY, USA;
3. The Force-Sensitive Resistor—the sensor Whadda WPSE477 [46] product by Velleman Group NV, Gavere, Belgium.

An FSR single point sensor was used the WPSE477 type with a 7.62 mm diameter sensing area, 0.2 mm thickness, measure range up to 0.5 kg (pressure threshold < 20 g), and a declared accuracy of ±2.5% (in the 85% range interval) [46]. The Si7021 chip can measure the relative humidity in the range of 0–80% RH with an accuracy of ±3%, and the temperature from −10 °C to +85 °C with a typical accuracy of ±0.4 °C [43].

**Figure 6:**
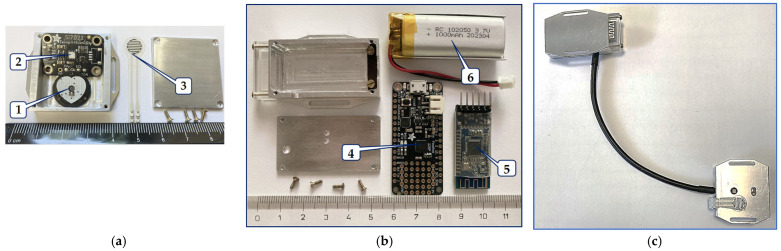
Construction of the PPGs-HTF sensor assembling: (**a**) photo of a measuring probe, where 1 = optical pulse sensor, 2 = humidity and temperature I2C sensor, and 3 = FSR pressure sensor, (**b**) photo of a sensor’s body including a Li-Po battery, where 4 = micro-controller board, 5 = BT-BLE communication module, and 6 = Li-Po battery, and (**c**) complete sensor with a patch cord without a Li-Po battery.

The sensor’s body is based on the Arduino-compatible micro-controller board Adafruit Feather 328P, by Adafruit Industries, New York, NY, USA, using the processor ATmega328P by Atmel Corporation, San Jose, CA, USA, running at 3.3 V logic and 8 MHz, with eight ten-bit ADCs, including also I2C hardware, and SPI support, a hardware USART-to-USB (CP2104) converter, and a charger for lithium polymer (Li-Po) batteries [47]. Here is also located the bi-directional communication BT module MLT-BT05, by Techonics Ltd., Shenzhen, China, working in the BT4.0 BLE standard at 2.4 GHz. The whole sensor is normally powered by the 3.7 V/1000 mAh Li-Po battery, but it is also possible to use the 5 V USB port for the purpose of micro-controller programming or debugging, and mainly for the charging of a battery cell. To enable measuring in a scanning area of a running MRI device, both parts of this wearable PPG multi-sensor consist of non-ferromagnetic components, and it is shielded by aluminum boxes—see assembling photos in Figure 6.

Using the bi-directional BT communication, the whole sensor works in the “slave” mode, waiting for commands from the master device, and as a response it sends the data records in the requested type, structure, and format. The service program on a micro-controller realizes the real-time sensing and digitalization of the analog PPG and FSR signal(s) with the chosen sampling frequency *f*_S_ in the range from 100 Hz to 1 kHz, and/or readings of the humidity/temperature values from the Si7021 chip in the chosen time interval *T*_INT_. Next, the data block with the length of *N*_MEAS_ = {1 k, 4 k, 16 k, 32 k, and 64 k} using 16-bit data samples is set for transmission to the control device. Finally, the number of channels used (from one to four) must be adjusted and the size of head communication data buffers must be set. On the side of the master device, the application created for Windows platform controls the acquisition of one or more-channel signals, their pre-processing, and data storage for future detailed analysis.

### 4.2. Auxiliary Experiments and Investigations

After verifying the sensor’s basic functionality in the normal laboratory conditions, three auxiliary measurements were performed:

1. Testing the stability of the wireless communication and the quality of BT connection between the sensor and the control laptop in three conditions:
  - MRI device is ready to scan, but no MR sequence is running—open shielding cage door (Cond1);
  - Cage door is closed, but without MR scanning (Cond2);
  - MR scan sequence is executed—the door must be closed (Cond3).
2. An investigation of the conductance/pressure conversion characteristics of the used FSR component by comparative measurement based on a set of calibration weights [48]. Then, the calculation of the linearization of the conversion characteristics for correct contact pressure determination.
3. The evaluation of the accuracy of the HR value determination process by comparative measurements being taken in the normal laboratory conditions, namely PPG and FSR signals sensing and measurement with an oximeter device in parallel.

**Figure 7:**
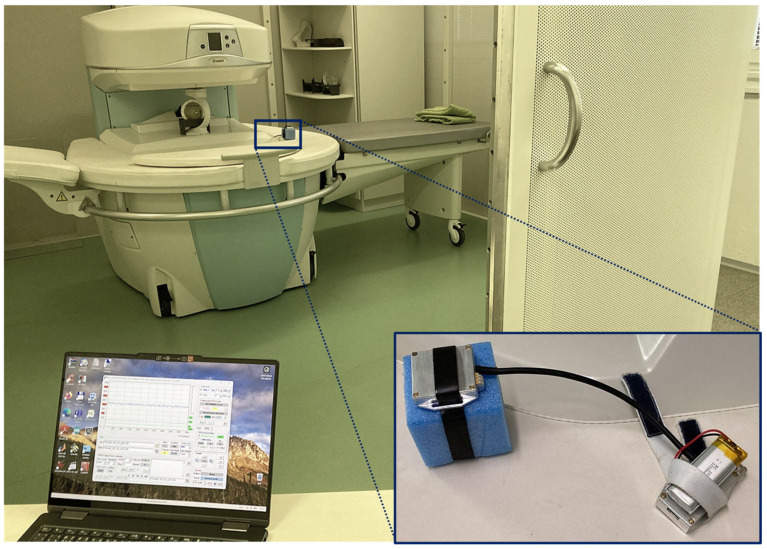
Arrangement of RSSI measurement inside the MRI device E-Scan Opera located in the metal shielding cage and a control laptop in front of the open cage door.

The first auxiliary investigation was performed in the low magnetic field environment of the open-air MRI device E-Scan Opera by Esaote S.p.A., Genoa, Italy, working with a static magnetic field of 0.178 T located at the Institute of Measurement Science, Slovak Academy of Sciences in Bratislava (IMS SAS). This device contains a gradient system consisting of three gradient coils that produce three orthogonal linear fields for spatial encoding of a scanned object [49]. The stepwise changed magnetic field and RF distribution present during the scanning process can affect the stability and quality of any wireless communication with the sensors inside and/or in the vicinity of the running MRI device. For this reason, it is necessary to test, verify, and evaluate the functionality and stability of the BT connection for each of the new, realized PPG sensors before its practical use in greater (long-time) measuring experiments. In addition, the whole MRI scanning equipment is placed in a metal cage to suppress high-frequency interference. The cage is made from a 2 mm thick steel plate with 2.5 mm diameter holes distributed periodically in a 5 mm grid to eliminate the propagation of the electromagnetic field to the surrounding space of the control room [49]. The tested multi-sensor was present inside the scanning area of the MRI device; the control laptop was situated outside the shielding cage—see the overall photo in Figure 7. During this measurement a testing spherical water phantom was placed in the RF receiving/transmitting coil, and no person was investigated. To obtain also a signal from the FSR component, the measuring probe was fixed on a plastic cube (simulating a human wrist) by a polyamide ribbon. The basic measurement was realized using the power supply by 3.7 V Li-Po battery (as documented by the detail in Figure 7). The second set of measurements for comparison was performed using the 5 V powering via USB port on the micro-controller board by a power bank. All measurements were realized at a distance between the sensor and the control device of about *D*x ≅ 2.5 m, and within an air-conditioned environment for the MRI equipment, with the mean temperature of 24 °C. In the frame of the third testing condition, the MR scan 3D-CE sequence was running [49]. The quality of the BT connection was evaluated by the received RSSI parameter representing an estimated measure of the power level received by a mean and the std RSSI values calculated from five measurements per each testing condition and two types of sensors’ power supply.

**Figure 8:**
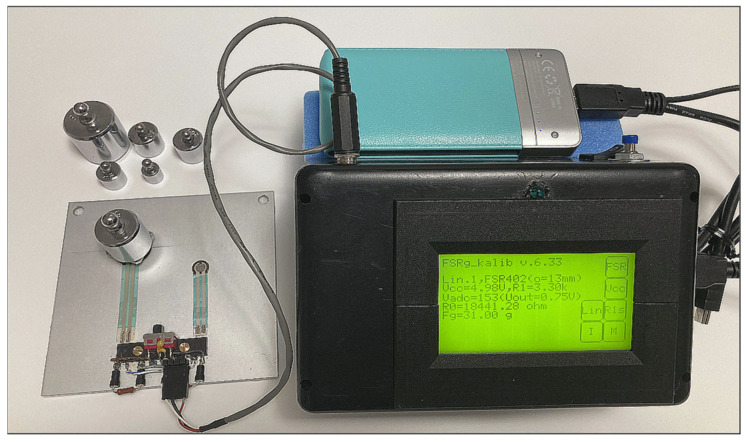
Documentary photo of the second auxiliary experiment—measurement of the FSR resistance/pressure characteristic using the previously developed semi-automatic measuring tool [48].

The second auxiliary measurement was realized in three phases. At first, the declared catalog resistance/pressure conversion characteristic of the tested FSR component was verified by comparative measurements using a previously developed semi-automatic measurement tool [48]. This tool is based on the Arduino micro-controller and it uses a programmable touch control panel for measuring in manual mode or the BT communication module for wireless control in the semi-automatic mode. In the manual mode, obtained discrete *V*_OUT_ and calculated *R*_FSR_, *G*_FSR_, and *F*_MEAS_ values are immediately shown on the display, without any storing or other processing. When the semi-automatic mode is chosen, the setting of measuring parameters and the real-time transmission of a digitized analog signal *V*_OUT_ are operated wirelessly from the control device. Received data blocks can be stored to an external file in the MS Wave format for further analysis, with the help of the Matlab signal processing and the statistical toolbox. The truncation of the applied pressure range arises from the real conditions of experiments with human subjects; measurements with applied 19 force values, corresponding to weights of *Fg* = {50, 75, 100, 125, 150, 175, 200, 225, 250, 275, 300, 325, 350, 375, 400, 425, 450, 475, and 500 g}, were performed only. For this purpose, a set of eight calibration weights (10 g–500 g) in the accuracy class M2 was used. The currently tested FSR element was fixed on an aluminum plate, and the testing weights (and/or their combinations) were placed on the FSR’s sensing area, as shown in a documentary photo in Figure 8. The whole measuring tool was supplied by a 5 V power bank with capacity of 20,000 mAh. The practical measurement of FSR characteristics was performed in two steps: (1) for each applied force, the corresponding *V*_OUT_ value was measured manually, and (2) in semi-automatic mode, 4-k sample data records of the sampled *V*_OUT_ signal at *f*_S_ = 125 Hz were received and stored. Next, the *R*_FSR_ (*F*g) relations and *G*_FSR_ conversion characteristics were calculated with the finally denoted linearization calculated by the mean square error method. The second phase of this auxiliary experiment consists of the linearization of depicted conversion characteristics based on the linear regression approach proposed for easier implementation into the PPG sensors’ control program. Finally, the linearization accuracy was evaluated. All performed measurements used a voltage divider with the push-down resistor *R*1 = 3.3 kΩ (Vcc = 5 V).

**Figure 9:**
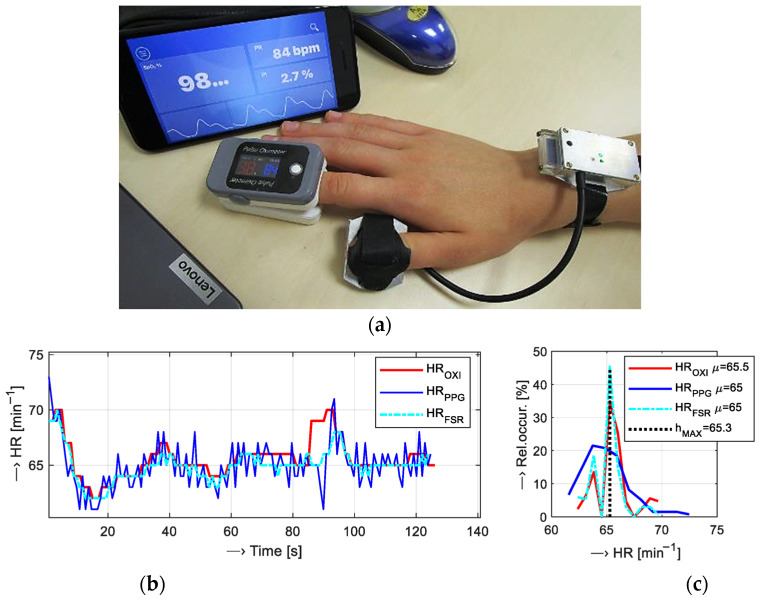
Comparative measurement of HR values based on the oximeter device: (**a**) arrangement photo, with the PPG multi-sensor probe worn on the thumb and oximeter on the forefinger of the right hand, (**b**) an example of the HR values received from an oximeter together with the values determined from PPG and FSR signals, and (**c**) calculated histograms together with mean values.

For the calibration of HR measurements, the pulse oximeter Berry BM1000 C [50], by Shanghai Berry Electronic Tech Co., Ltd. (Shanghai, China), was used. This type of oximeter, with an optical sensor working in a transmission mode, enables the display of calculated arterial blood oxygen saturation, HR, and perfusion index values, which are simultaneously transmitted to the control device (tablet, smartphone, etc.) via BT connection. An Android/iPhone application running on the master device stores the received data to an internal memory with the possibility to export them in the *.csv format for further off-line processing. The measuring probe of the multi-sensor was mounted on the thumb (while the sensor’s body was on the wrist) and the oximeter device on the forefinger, as documented in Figure 9a. The recording of the PPG signals as well as the oximeter values lasted for 128 s. The synchronization of both parallel measurements was realized manually. The achieved HR values in *.csv file also contain the timestamps. The files of the recorded PPG and FSR signals include time information about the start of the sensing operation, so the actual position of the determined HR value is given by a localized systolic pulse in the samples (see the demonstration example in Figure 1b) and the chosen sampling frequency *f*_S_. For this reason, the time correlation between HR sequences from the oximeter and the PPG multi-sensor can be easily guaranteed. Data obtained in this way were next processed and analyzed—see an example of the visualization of the HR values received from an oximeter together with the values determined from the PPG and FSR signals in Figure 9b, and the subsequently calculated histograms of data distribution together with enumerated mean values in Figure 9c. Then, differential sequences *HR*_DIFF_ were calculated and analyzed statistically. Finally, the MAE values for the *HR*_PPG_ and *HR*_FSR_ sequences were enumerated. In this part of auxiliary measurements, six tested subjects (three males and three females) were joined. All these people (TP_1,2,8M_, TP_1,2,4F_) also participated in the main measurement experiment.

### 4.3. The Main Measurement Experiment

**Figure 10:**
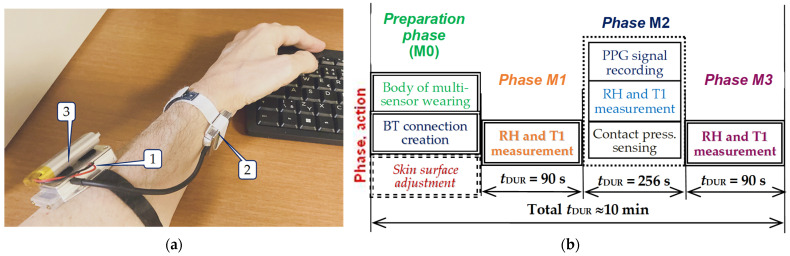
Realization of main measurement experiment: (**a**) principal arrangement photo, where 1 = sensor’s body, 2 = measuring probe, and 3 = Li-Po battery, and (**b**) applied experimental time schedule.

After the auxiliary investigations, the main measurement experiment was performed. It consisted of the real-time sensing of PPG waves, and humidity and temperature values, together with information about the current contact pressure applied on the skin surface by a measuring probe. The tested person sat with a hand laid on a table in a normal office room (with the mean temperature of about 24 °C) without any additional stimuli. The measuring probe was worn on the wrist artery of the left or right hand, while the sensor’s body was fixed on the upper part of the arm, as demonstrated by the arrangement photo in Figure 10a. The measurements were realized for three skin moisture levels:

- The sensor’s probe was worn without any adjustment to the skin surface of the bottom wrist area (Normal);
- The skin surface was dried by a hairdryer fanning cold air (Dry);
- The skin was partially moistened by a wet cloth before the probe wearing (Wet).

The principal experiment schedule drawn in Figure 10b shows that the real-time measurements were performed in the following phases:

1. In the preparation phase M0, without any practical measurement, when the body of the PPG multi-sensor is mounted on the tested person’s arm, the BT connection with the control device is established, and the quality of the sensed PPG and pressure (FSR) signals are verified. Next, some adjustment of the skin surface is performed depending on the required skin moisture level.
2. In the initial 90 s measurement phase M1, when the RH and T1 values are taken in the intervals of *T*_INT_ = 1 s. In the first 30 s of this duration, the measuring probe is freely laid on the desk (current air conditions are measured) and then the probe is put on the left/right wrist of the tested person. At this moment, the offset of RH and T1 sequences (*RH*_OFS_ and *T1*_OFS_) are determined.
3. In the phase M2 with the time duration of 256 sec, the PPG and FSR analog signals (sensed using the sampling frequency *f*_S_ = 125 Hz) together with RH and T1 values (with *T*_INT_ = 0.2 s intervals) are recorded in parallel.
4. In the final 90 s measurement phase M3, which starts at the moment the measuring probe is taken off of a wrist and the final HR and T1 values (*RH*_FIN_ and *T1*_FIN_) are received for Δ*RH*_PON_ and Δ*T1*_PON_ parameters’ calculation. In this case, the RH and T1 values are again recorded using *T*_INT_ = 1 s.

The total time duration of the whole measurement is approximately 10 min, depending on the length of the preparation part M0. The time duration of this phase F0 was strictly individual; practically, it depends on the actual volume of the wrist of the tested person, on the successfulness of finding the right position of the FSR element on the vessel to obtain the proper signal, and finally on the time taken to adjust the pressure force level by a fixation rubber (different for male and female subjects). Contrariwise, the skin manipulation (drying/moistening) operation was applied for 1 min in all cases. The total number *N*_HP_ of the detected heart periods usable for calculation of HR values (*HR*_PPG_ and *HR*_FSR_) depends on the actual state of the tested person during the measurement phase M2.

In the frame of main experiments, a small database of PPG signals and supplementary data records was collected. It originated from fourteen volunteers (eight males TP_1M_-TP_8M_ and six females TP_1F_-TP_6F_) with a mean age of 47 ± 18 years. All tested persons were without any important problems with a cardio-vascular system (no drugs applied), and except for the female subject TP_5F_, all remaining participants were right-handed. Six files per person—two from the M1 and M3 phases and one from the phase M2, picked-up on the left and right hands (6 × 12 = 72 in total)—were stored in the collected database. The sensed signals and data were subsequently processed off-line to determine the PPG and FSR signal properties and the changes in the RH and T1 sequences within all measuring phases, M1–3. The partial and summary results obtained for all tested persons were evaluated separately and, depending on the skin moisture levels, they were next analyzed to obtain their basic statistical parameters for the summary comparison.

As there exists principal differences between the skin properties of male and female persons, different contact pressure must be applied to obtain a proper signal from the FSR element that is suitable for HR values’ detection and their comparison with the values obtained from the PPG signal (comparable with the case shown in Figure 5b). In correspondence with our previous research [51], we finally tried to apply the mean contact pressure of *μ* CP = 100 g for the tested male subjects and *μ* CP = 75 g for the females.

## 5. Results

In the frame of the first auxiliary measurement, the mean and std values of the received RSSI parameter were obtained for two types of sensors’ power supplies, which are enumerated in Table 1.

**Figure 11:**
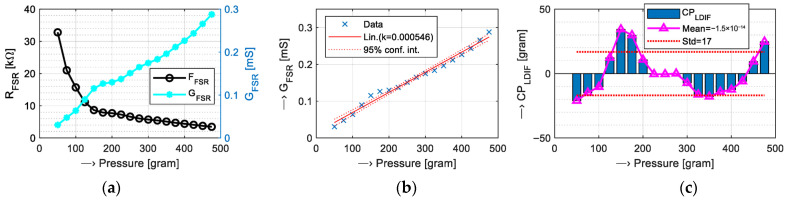
Linearization of conductance/pressure conversion characteristics: (**a**) *R*_FSR_ and *G*_FSR_ curves, (**b**) linearization for the whole range of *CP* = <50 ÷ 500> grams with denoted 95% confidence intervals, and (**c**) resulting *CP_LDIF_* values with denoted mean and std values; *R*1dwn = 3.3 kΩ, Vcc = 5 V.

The results of the second auxiliary measurement are presented in the form of *R*_FSR_ and *G*_FSR_ curves in Figure 11a. Applying the linear regression method on the measured data, a linearized conversion characteristic was proposed, as shown in Figure 11b. The results of the final accuracy of the performed linearization are also presented graphically—see Figure 11c.

**Figure 12:**
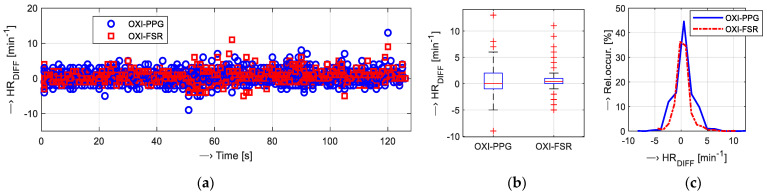
The summary results of calibration measurements: (**a**) visualization of concatenated *HR_DIFF_* values calculated from the *HR*_PPG_ (OXI-PPG) and *HR*_FSR_ (OXI-FSR) sequences, (**b**) box plot of basic statistical parameters of *HR*_DIFF_ values, and (**c**) histograms of *HR_DIFF_* values; all participants, left and right hands together.

The summary results of the calibration experiment, when the HR values determined from PPG and FSR signals were compared with the HR values measured by the pulse oximeter, are shown graphically in Figure 12, and the MAE values are enumerated in Table 2.

**Figure 13:**
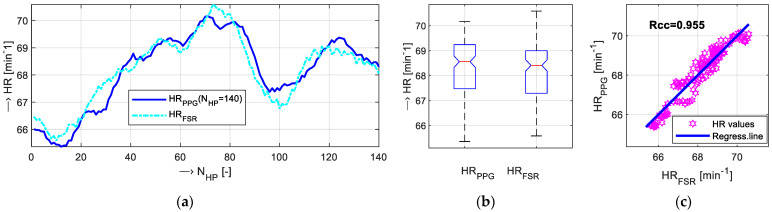
Comparison of *HR*_PPG_ and *HR*_FSR_ values: (**a**) visualization of *HR*_PPG_ and *HR*_FSR_ sequences, (**b**) box plot of basic statistical parameters, and (**c**) scatter plot together with regression line and denoted Pearson correlation coefficient *Rcc*; signals taken from the right hand of a male testing subject TP_1M_ after skin drying.

**Figure 14:**
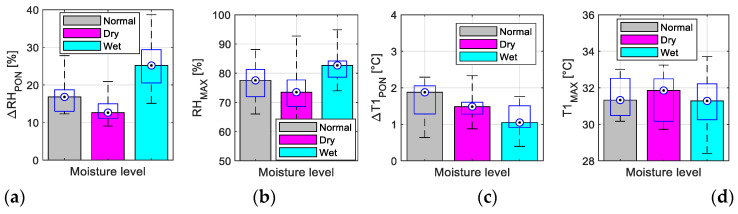
Summary results of the analysis of the relative humidity and temperature sequences for three skin moisture levels: bar graphs of mean values overlaid with box plots of basic statistical properties for (**a**) Δ*RH*_PON_, (**b**) Δ*T1*_PON_, (**c**) *RH*_MAX_, and (**d**) *T1*_MAX_.

**Figure 15:**
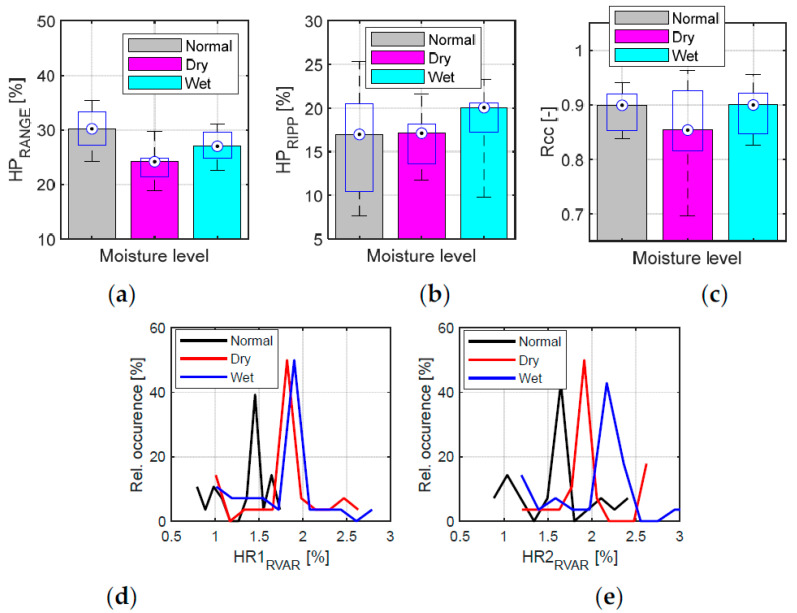
Tracking changes in PPG signal properties: (**a**) bar graphs overlaid with box plots of basic statistical properties of *HP*_RANGE_, (**b**) *HP*_RIPP_, (**c**) *R*cc, and (**d**) histograms of HR relative variance for HR1 from *HR*_PPG_ and (**e**) for HR2 from *HR*_FSR_.

**Figure 16:**
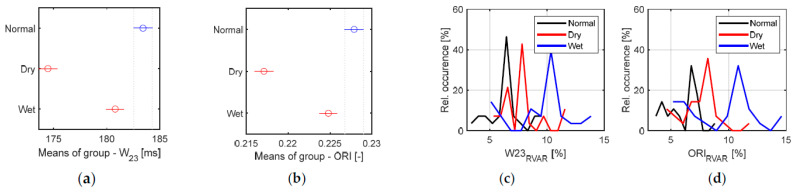
Summary results of ORI features: (**a**) graphs of multiple comparisons of group means applied to the results of ANOVA statistics for *W*_23_ values, and (**b**) for the *ORI* parameter, and (**c**) histograms of relative variances for *W*_23_, and (**d**) for ORI parameter.

**Figure 17:**
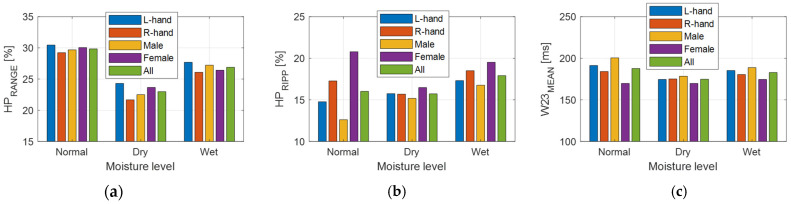
Detailed comparison per the type of the used hand (left/right) and the gender of the tested subject (male/female), and the summary results (all) for parameters with pronounced differences in correspondence, with applied skin manipulation giving three skin moisture levels; mean values of (**a**) *HP*_RANGE_, (**b**) *HP*_RIPP_, and (**c**) *W*_23_.

The comparison of the sequences of the HR values determined from the PPG and FSR signals taken from the right hand of a tested male subject, TP_1M_, after skin drying is shown in Figure 13a, the box plot of basic statistical properties can be seen in Figure 13b, and the scatter plot describing the correlation between the *HR*_PPG_ and *HR*_FSR_ sequences together with the calculated *Rcc* coefficient is presented in Figure 13c. Table 3 gives the partial results for all three tested skin moisture levels. The presentation of the summary results of the main experiment is structured as follows:

1. The results of the analysis of the relative humidity and temperature sequences for three skin moisture levels—see the bar graphs of the mean values together with box plots for Δ*RH*_PON_ and Δ*T1*_PON_, and *RH*_MAX_ and *T1*_MAX_ parameters in Figure 14.
2. Tracking changes in PPG signal properties—see the bar graphs of *HP*_RANGE_, *HP*_RIPP_, and HR relative variance (HR1 from *HR*_PPG_; HR2 from *HR*_FSR_), and Rcc in Figure 15.
3. The comparison of the basic statistical properties of ORI-based parameters—see histograms and bar graphs of mean and variance for *W*_23_ and *ORI* in Figure 16.
4. Detailed analysis of three most important parameters (that obtained well-pronounced differences in correspondence with applied skin manipulation), categorized by the type of the used hand and the gender of the tested person separately—see the bar graphs of *HP*_RANGE_, *HP*_RIPP_, and *W*_23_ in Figure 17.

The summary description of the detected numerical trends in all analyzed parameters is given in Table 4. Using the “Normal” moisture level as a reference value, the absolute and relative percentual differences for the “Dry” and “Wet” levels in the selected compared parameters are enumerated in Table 5.

## 6. Discussion

The performed measurement confirms the functionality of the developed special prototype of a wearable multi-sensor, enabling the measurement of PPG signals, relative humidity, temperature values, and contact pressure by an FSR element. Auxiliary experiments check the practical possibility of wireless BT connection and data transfer through the shielding cage of the open-air MRI device, but with lower signal intensity. The minimum RSSI of –94 dBm was reached when the cage door was closed and an MR scan sequence was running for both types of sensors’ power supplies. At present, higher-quality BT BLE receivers have a sensitivity of more than −100 dBm, so the obtained minimum RSSI value still guarantees the proper stability of the BT communication. On the other hand, the performance of the actual connection also depends on the sensitivity of the BT transmitter/receiver used on the side of the control device (laptop/tablet, etc.). Differences in the RSSI values using 3.7 V or 5 V supplying were inconsiderable, as documented by the mean values in Table 1. In all cases of RSSI measurements, the real-time data transfer via BT connection was functional (error free), and the data received from analog signals were usable for further processing and analysis. For normal experimental use, when the PPG multi-sensor is worn by the tested person lying inside the scanning area of the MRI device, we suppose the powering by a Li-Po battery. For testing or calibration purposes only, the 5 V supply could also be applied.

The initial resistance of the FSR element is typically > 100 MΩ (no load), while for the maximum pressure, *R0* is about 2.5 kΩ (depending on the FSR type) [44]. Therefore, as a compromise choice, the setting of *R1* = 3.3 kΩ was finally used in the main investigation experiment. While the tested FSR element can measure pressures up to 0.5 kg, only the lower part up to 250 g will be practically used to prevent possible pain or other negative effects on the human testing subjects. The previously performed analysis of the influence of the used push-down resistor *R1* on the measured *G*_FSR_ characteristics has shown that higher *R1* decreases the *G*_FSR_ nonlinearity, but also the dynamic range (sensitivity) [48]. Based on the linear regression approach used for one-part linearization of the pressure/conductance characteristic, the finally obtained mean difference Δ*CP*_MEAN_ was near zero, while the partial maximum Δ*CP*_MAX_ was about 20 g and the summary Δ*CP*_STD_ was ±17 g (see the right graph in Figure 11c), which is near the declared pressure threshold [46] and fully sufficient for our current experimental purpose.

Comparative measurements with the help of the oximeter device show good correspondence with HR values determined from PPG and FSR signals. The final MAE values calculated from *HR*_PPG_ (OXI-PPG) and *HR*_FSR_ (OXI-FSR) sequences presented in Table 2 show that the mean *HR_DIFF_* for both of the tested HR determination approaches have an acceptable and similar accuracy of about 0.5 min^−1^ and std which lies below 2 min^−1^. Histograms calculated from all performed comparative measurements in Figure 12c document that distributions of *HR_DIFF_* values are similar in both cases. The detailed statistical analysis yields slightly higher variance of the OXI-PPG-type values in comparison with the OXI-FSR type. Next, there are no essential differences between the HR values determined from the signals sensed on the left versus the right hands; PPG and FSR signals from the left hand produce a slightly higher MAE (compare 0.59 vs. 0.37 for OXI-PPG) but with a smaller variance (see std of 1.29 vs. 1.49 for OXI-FSR).

From the results of the realized main measuring experiments it follows that the performed skin manipulation (skin drying and/or moistening) was detectable in all cases, as documented by changes in the Δ*RH*_PON_ parameter in Figure 14a. As it can be seen here, when the moistening was applied, the highest Δ*RH*_PON_ values were always achieved. From the point of view of the Δ*T1*_PON_ parameter (see Figure 14c), the situation is the opposite: skin moistening produces the smallest values. Different skin moisture levels are mainly expressed by changes in *HP*_RANGE_ means, as documented by the graphical results in Figure 15a. With regard to HR variances, HRs determined from the FSR signal have higher *HR2*_RVAR_ values as documented by histograms in Figure 15d,e. The histograms of variations in *W*_23_ and ORI contain different clearly expressed maxima of relative occurrence—see Figure 16c,d. Skin drying decreases the *HP*_RANGE_, while moistening increases the PPG signal ripple. The smallest *R*cc values (*HR*_PPG_ and *HR*_FSR_ differing mostly) were obtained after the skin drying operation (see the bar graph in Figure 15c). The highest variances of the HR values, and *W*_23_ and ORI parameters are achieved when the skin moistening was applied, as can be seen in Figure 16c,d. The multiple comparisons of group means applied via the ANOVA statistics for the *W*_23_ and ORI parameters confirm that their values for all three moisture levels are statistically separable. The visualization in Figure 16a,b shows that all three groups, “Normal”, “Dry”, and “Wet”, are fully different and separable.

From the detailed analysis performed separately per hand and gender types it follows that there exist differences in the *HP*_RANGE_ and *W*_23_ parameters (which are mostly expressed changes caused by skin manipulation) between the results of male and female subjects tested. On the other hand, the results of the measurements on the left and right hands differ less (not very significantly), as can be seen in bar graphs in Figure 17. The situation is more complex in the case of *HP*_RIPP_ parameter—here the obtained values differ significantly for the gender as well as the hand type.

The performed summary enumeration of percentage differences in selected parameters for the “Dry” and “Wet” skin moisture levels relative to the “Normal” level brought the following quantitative findings. The application of skin moistening increases the mean humidity by 14% while drying decreases the humidity by 8%. Temperature changes were negative in both cases—decrease by 45% for moistening and 21% for drying (absolute changes in °C were –0.39 and –0.84). This was caused by the fact that, for drying, a hairdryer fanning cold air was used and, for moistening, an applied cloth was wetted by cold water. In comparison with the normal skin state, drying decreases the PPG signal range by 7% and the systolic pulse width by 8%, while moistening caused an increase of 3% in the signal ripple, a decrease in the correlation between heart rate values *HR*_PPG_ and *HR*_FSR_ by 5%, and a decrease in the ORI by 3%.

## 7. Conclusions

The developed special prototype of a wearable PPG multi-sensor enables the real-time sensing of the skin moisture and the temperature as well as measurement of the physical contact pressure between the sensor’s probe and the skin surface in the magnetic field environment existing in the MRI equipment, working with a low magnetic field up to 0.2 T. The practical functionality of the realized multi-sensor was verified in the scanning area of the open-air MRI tomograph, but without using the human testing subject, at this moment. An additional comparative measurement with a certified commercial oximeter device shows good agreement between the determined HR values.

The implemented FSR element is also practically usable to sense heart pulsation on the wrist radial artery as an alternative approach when the sensed PPG signal is degraded or the systolic pulses have very small amplitude, so it is not possible to determine correctly the HR, ORI, and other parameters, or to estimate ABP values with sufficient precision. Last but not least, the FSR sensor will be used to validate correct setting of the contact pressure for the entire measuring probe. It is necessary to exclude situations where too much contact force compresses and declines the PPG signal taken from the vessel.

The performed testing confirms that unlike the mainly used sensors, based on skin electrical conductance, the implemented air humidity-measuring component for non-contact skin moisture investigation using the FSR measurement principle is fully applicable in the low magnetic environment with RF and electro-magnetic disturbance. Relative humidity values obtained before starting and during the PPG signal measurement can be used to assess the final validity of the measuring conditions for each of the currently performed long-term experiments. First of all, it is necessary to detect (check) these values to avoid limit states, e.g., skin that is too dry or, on the contrary, extremely moisturized skin, which (>90% RH) cannot provide a usable PPG signal for further processing.

The basic limitation of this work is that relatively few measurements, on a small group of tested persons, were made, and a small corpus of collected PPG, humidity, temperature, and contact pressure data records was processed and analyzed. In this way, the obtained results of these first-step experiments cannot be generalized and correctly compared with the works of other researchers. Despite this fact, our working premise about the evocation of changes in determined PPG wave features via the application of different skin moisture levels was successfully verified. The second limitation lies in the fact that the MRI devices located at the IMS SAS (one open-air and one whole-body tomograph) cannot be used for clinical medical research, because our institute is not certified for work with real patients. For this reason, the testing subjects participating in measurements were typically the authors themselves and their close colleagues from the IMS SAS. A possible way of solving this limitation is to establish some cooperation with the nearest medical centers in Bratislava (Slovakia) or Vienna (Austria).

In the near future, we plan to realize measuring experiments directly inside the running (scanning) MRI device(s) located at our institute and in this way to collect a larger database consisting of PPG and contact pressure signal records, together with humidity and temperature values. Subsequently, we would like to use the determined changes in the skin humidity and temperature for detection, classification, and quantification of the physiological stress induced by the examination process in the real clinical MRI devices in cooperation with the mentioned medical centers.

## Figures and Tables

**Table 1:** Mean and std RSSI values measured in three conditions inside the E-Scan Opera device.

Powering ^(A)^/Condition	Cond1	Cond2	Cond3
3.7 V Li-Po battery	–86.2 ± 0.3 dBm	–92.3 ± 0.5 dBm	–93.7 ± 0.8 dBm
5 V power bank	–85.8 ± 0.5 dBm	–92.1 ± 0.6 dBm	–93.6 ± 0.7 dBm

^(A)^ The BT BLE module working in BT 4.1 standard; serial communication operated at 57,600 bps.

**Table 2:** Final MAE values for *HR*_PPG_ (OXI-PPG) and *HR*_FSR_ (OXI-FSR) sequences.

*HR_DIFF_* ^(A)^	L-Hands (min^−1^)	R-Hands (min^−1^)	All (min^−1^)
OXI-PPG	0.59 ± 1.98	0.37 ± 2.01	0.45 ± 1.84
OXI-FSR	0.61 ± 1.29	0.41 ± 1.49	0.51 ± 1.40

^(A)^ All data records with duration of 128 s.

**Table 3:** Partial results of PPG signal properties and HR statistical features for three skin moisture levels; right hand of the subject P1_M_.

Condition ^(A)^	*HP*_RANGE_ [%]	*HP*_RIPPLE_ [%]	*HR1*_RVAR_^(B)^ [%]	*HR2*_RVAR_^(C)^ [%]	*Rcc* [−]	*W*_23_ [ms]	*ORI* [−]
Normal	26.8	21.8	0.83	1.10	0.806	210	0.235
Dry	18.3	16.0	1.65	1.84	0.955	156	0.176
Wet	25.7	25.4	1.38	1.58	0.872	184	0.195

^(A)^ Measuring probe put on with the applied CP ≅ 100 g. ^(B)^ Calculated from *HR*_PPG_. ^(C)^ Calculated from *HR*_FSR_.

**Table 4:** Detected trends in changes in PPG signal properties for three investigated skin moisture levels; summary results of the analysis using all collected data records.

Parameter/Moisture Level	Normal	Dry	Wet
*HP*_RANGE_	higher	smallest	~
*HP*_RIPP_	similar	similar	highest
*HR1*_RVAR_	smallest	~	higher
*HR2*_RVAR_	smallest	~	higher
*Rcc*	~	smallest	higher
*μ W*_23_	higher	smallest	~
*W23_RVAR_*	smaller	~	highest
*μ ORI*	higher	~	smallest
*ORI_RVAR_*	smaller	~	highest

**Table 5:** Absolute and relative percentage differences in selected parameters for “Dry” and “Wet” skin moisture levels based on the “Normal” level as a reference.

Parameter/Moisture Level	Normal–Dry		Normal–Wet	
	*Abs*	*Rel*	*Abs*	*Rel*
Δ*RH*_PON_ [%]	–8.4	~	14.2	~
Δ*T1*_PON_ [°C]	~	–20.9	~	–45.1
*HP*_RANGE_ [%]	–6.9	~	–2.5	~
*HP*_RIPP_ [%]	0.1	~	3.1	~
*HR1*_RVAR_ [%]	0.7	~	0.8	~
*HR2*_RVAR_ [%]	0.5	~	1.1	~
*Rcc* [−]	~	–4.9	~	0.2
*μ W*_23_ [ms]	~	–8.4	~	–2.1
*W23_RVAR_* [%]	1.00	~	2.9	~
*μ ORI* [−]	~	–2.2	~	–3.4
*ORI_RVAR_* [%]	3.02	~	3.91	~

## Data Availability

The raw data supporting the conclusions of this article will be made available by the authors on request.

## Abbreviations

The following abbreviations are used in this manuscript:

**Abbreviation**	**Definition**
ABP	Arterial blood pressure
ADC	Analog-to-digital converter
ANOVA	Analysis of variances
BT	Bluetooth
CP	Contact pressure
ECG	Electrocardiography
FD-PPG	First-derivative PPG
FID	Free induction decay
FSR	Force-sensitive resistor
GSR	Galvanic skin response
HP	Heart pulse
HR	Heart rate
HRV	Heart rate variability
IMS SAS	Institute of Measurement Science, Slovak Academy of Sciences
Li-Po	Lithium polymer
MAE	Mean absolute error
MRI	Magnetic resonance imager
ORI	Oliva–Roztocil index
PPG	Photoplethysmography
PTT	Pulse transmission time
PWV	Pulse wave velocity
RF	Radiofrequency
RH	Relative humidity
RSSI	Received signal strength indicator
SD-PPG	Second-derivative PPG
TE	Echo time
TR	Repetition time
